# Next generation targeted non-genotoxic conditioning for hematopoietic stem cell and hematopoietic stem cell-based gene therapy

**DOI:** 10.3389/fimmu.2025.1653344

**Published:** 2025-10-01

**Authors:** Jennifer Okalova, Trent H. Spencer, Shanmuganathan Chandrakasan

**Affiliations:** ^1^ Department of Pediatrics, Emory University School of Medicine, Atlanta, GA, United States; ^2^ Aflac Cancer and Blood Disorders Center, Children’s Healthcare of Atlanta, Atlanta, GA, United States; ^3^ Molecular and Systems Pharmacology Program, Graduate Division of Biological and Biomedical Sciences, Laney Graduate School, Emory University, Atlanta, GA, United States

**Keywords:** HSCT, gene therapy, non-genotoxic, conditioning, transplantation, HSC niche

## Abstract

Hematopoietic stem cell transplant (HSCT) and hematopoietic stem cell (HSC)-based gene therapy, including gene editing approaches, offer a promising strategy for addressing numerous lymphohematopoietic genetic defects. Although significant progress has been made since the first HSCT over 60 years ago, the widespread application of allogeneic HSCT and autologous gene therapy is still hindered by the need for pre-transplant conditioning. The eradication of host HSCs and their progeny is widely thought to be necessary to create “space” in the bone marrow niche and enable long term engraftment of transplanted cells. However, despite decades of research, alkylating agents such as busulfan, melphalan and treosulfan or total body irradiation still remain the backbone of most HSCT condidtioning regimens. These genotoxic conditioning agents are non-targeted and leave patients susceptible to infections, infertility, organ toxicities, and secondary malignancies. As a result, there is an urgent need to develop alternative, non-genotoxic conditioning regimens that can selectively deplete HSCs while sparing cells outside the lymphohematopoietic compartment. A growing body of preclinical and clinical breakthroughs demonstrate the effectiveness of monoclonal antibodies, antibody-drug conjugates, immunotoxins, radioimmunotherapy compounds, and even T cell redirection strategies for achieving targeted HSC elimination. The use of these new agents can transform HSCT, and in this review we aim to highlight the potential and limitations of next-generation, non-genotoxic or minimally toxic conditioning methods. These alternatives to conventional chemoradiation could reduce toxicity and improve the safety of HSC-based gene therapies, ultimately expanding patient access and eligibility for these transformative treatments.

## Introduction

The unique ability of hematopoietic stem cells (HSCs) to differentiate into multiple lineages and self-renew has facilitated the development of curative strategies such as allogeneic hematopoietic stem cell transplant (HSCT) and autologous HSC-based gene addition/editing approaches to treat genetic defects in the lymphohematopoietic compartment. However, HSCs are a rare population, with a frequency of only 1 in 10,000 bone marrow (BM) cells, and the majority of long-term HSCs remain quiescent (1). These HSCs are sustained by a specialized BM niche composed of a diverse array of cell types, including mesenchymal stromal cells, endothelial cells, osteolineage cells, non-myelinating Schwann cells, and other hematopoietically derived cells, which collectively support and maintain HSC function (2). This quiescent and well nurtured rare population of HSCs can be classified as either long-term (LT) or short-term (ST) depending on self-renewal potential. Hematological stressors activate the LT-HSCs with extensive self-renewal potential to differentiate into ST-HSCs which are committed to multilineage differentiation into the downstream progenitors (3).

HSCT has been the primary definitive treatment modality for genetic defects in the lymphohematopoietic compartment. However, in allogeneic HSCTs there is an inherent risk of graft versus host disease (GVHD) and a limited availability of human leukocyte antigen-matched donors. Therefore, in hopes of reducing the risk of GVHD and decreasing the toxicity of allogeneic HSCT, autologous HSC-based gene addition and gene-editing strategies are now being developed. Despite encouraging results from preclinical studies and the clinical trials depicted in **Table 1**, both allogeneic and autologous HSC-based gene therapy strategies remain limited by the requirement for the patient to undergo conditioning prior to transplant to create adequate “space” in the BM niche for the homing of incoming allogeneic HSC or genetically-modified HSCs.

**Table 1:** Select clinical examples of conditioning agent and regimen intensity utilized in hematopoietic stem cell based gene addition/editing studies.

Disease	Study ID	Gene therapy	Agent(s)	Intensity	Busulfan target AUC/dose
Immune deficiency					
ADA	NCT00598481	γ-RV	Busulfan	Nonmyeloablative	19.2-22.4 mg*h/L
ADA	NCT02999984	LV	Busulfan	Nonmyeloablative	20 mg*h/L
X-SCID	NCT01512888	LV	Busulfan	Nonmyeloablative	22 mg*h/L
X-SCID	NCT03311503	LV	Busulfan	Nonmyeloablative	30 mg*h/L
Artemis	NCT03538899	LV	Busulfan	Nonmyeloablative	20 mg*h/L
CGD	NCT02234934 NCT01855685	LV	Busulfan	Myeloablative	70–75 mg*h/L
WAS	NCT01515462	LV	Busulfan Rituximab* Fludarabine*	Nonmyeloablative	48 mg*h/L
LAD-1	NCT03812263	LV	Busulfan	Myeloablative	65 mg*h/L
Metabolic disorder					
MLD	NCT01560182	LV	Busulfan	Myeloablative	85 mg*h/L
Hurler	NCT03488394	LV	Busulfan	Myeloablative	85 mg*h/L
Hemoglobinopathy					
SCD	NCT02140554	LV	Busulfan	Myeloablative	59–82 mg*h/L,
SCD	NCT02186418	LV	Melphalan	Myeloablative	140 mg /m^2^ (Melphalan)
SCD	NCT03745287	CRISPR-Cas9	Busulfan	Myeloablative	80–100 mg*h/L
Thalassemia	NCT02906202	LV	Busulfan	Myeloablative	66–82 mg*h/L
Thalassemia	NCT03655678	CRISPR-Cas9	Busulfan	Myeloablative	80–100 mg*h/L
Bleeding disorder					
HA	NCT04418414	LV	Treosulfan Fludarabine* ATG*	Myeloablative	42 mg/kg (Treosulfan)
HA	NCT03818763	LV	Melphalan Fludarabine	Nonmyeloablative	120 mg /m^2^ (Melphalan)

Data taken from searches conducted for clinical studies at https://www.clinicaltrials.gov based on hematopoietic stem cell gene therapy. Columns list disease categorization, study ID, gene therapy, conditioning agent(s), regimen intensity, and then the specific area under the concentration-time curve (AUC) of busulfan for clinical outcome. Asterisk denotes additional conditioning agents employed in cases where a multi-agent regimen is applicable. ADA, adenosine deaminase deficiency; X-SCID, X-linked severe combined immunodeficiency; CGD, chronic granulomatous disease; WAS, wiskott-aldrich syndrome; LAD-1, leukocyte adhesion deficiency type 1; MLD, metachromatic leukodystrophy; SCD, sickle cell disease; HA, hemophilia A; γ-RV, gamma-retroviral vectors; LV, lentiviral vector; CRISPR-Cas9, clustered regularly interspaced short palindromic repeats and CRISPR-associated protein 9.

Traditionally highly potent alkylating chemotherapy agents, or irradiation, are utilized to enable engraftment of transplanted healthy allogeneic or genetically-engineered autologous HSCs. However, as will be discussed in this review, these non-targeted and DNA damaging agents do not just limit their depletion to solely the LT/ST-HSCs, but deplete the entire hematopoietic stem and progenitor cell (HSPC) compartment. Since conditioning eradicates the recipient HSPCs which saturate the BM niche in steady state, this depletion provides space for the transplanted cells to home and engraft (4). HSPC populations can also expand substantially in their native environment and maintain a coordinated balance between quiescence and activation. Therefore, in addition to depletion, these conditioning regimens also serve the benefit of stimulating the BM niche to release cytokines and produce factors that promote engraftment (5, 6). Finally, the intent of a conditioning regimen is not solely myeloablation. These conditioning agents are also used to achieve immunosuppression to prevent rejection of the donor HSPC graft in allogeneic HSCTs or to suppress the immune response to a transgene in some genetically-engineered autologous HSC-based gene therapies.

The choice of conditioning regimen depends on both the patient’s disease and source of HSCs, and is classified as myeloablative (MA), reduced intensity conditioning (RIC), or non-MA (NMA). The categorization of these conditioning regimens depends on endogenous BM ablation, stem cell support, and degree of cytopenia imposed by the regimen (7, 8). Taking these varying levels of intensity into consideration, patients subject to MA conditioning will undergo irreversible pancytopenia and require stem cell support compared to RID and NMA regimens. These regimens need to be carefully tailored to their specific disease setting where there are different extents of progenitor depletion required. For example, extensive HSPC clearance may be desired in disease states like leukemia where the conditioning will eliminate any residual pathogenic clones. There may also be benefits to this in the context of primary immunodeficiencies where the high level of clearance could also help overcome host progenitor competition. Since different conditioning regimens that are currently in use have variable organ toxicity profiles and myeloablation/immune ablation properties, it is critical for the intensity and selection of agents to be tailored to the disease setting to provide patients with the most ideal risk-benefit ratio.

Since the first HSCT was carried out in 1956 by E. Donnall Thomas, MA conditioning regimens remain the gold-standard for HSCT and utilize alkylating agents such as busulfan, treosulfan, melphalan, and thiotepa (8, 9). Collectively though the field has transitioned away from total body irradiation (TBI) and more towards multi-agent conditioning regimens to achieve myeloablation. Alkylating agents like busulfan are even now being personalized in the regimens using patient-specific clearance parameters to pharmacokinetically reduce target exposure, which is reflected in an area under the plasma concentration-time curve (10) (**Table 1**). These multi-agent conditioning regimens frequently require immune ablation in addition to the myeloablation to fully or partially suppress the immune system to decrease graft rejection, GVHD, or an immune response to transplanted genetically modified cells. **Table 1** highlights some gene therapy strategies where additional immunoablative agents are added to overcome underlying immune dysregulation or the immunogenicity of the transgene like in wiskott-aldrich syndrome and hemophilia A, respectively. These MA regimens include immunosuppressants like the antimetabolite fludarabine or for the patient to receive serotherapy with anti-thymocyte globulin or alemtuzumab to achieve selective depletion of mature lymphocytes. However, in addition to this immunosuppression it has been shown that some of these agents like fludarabine can also synergize in the regimens to maximize depletion (11).

**Figure 1:**
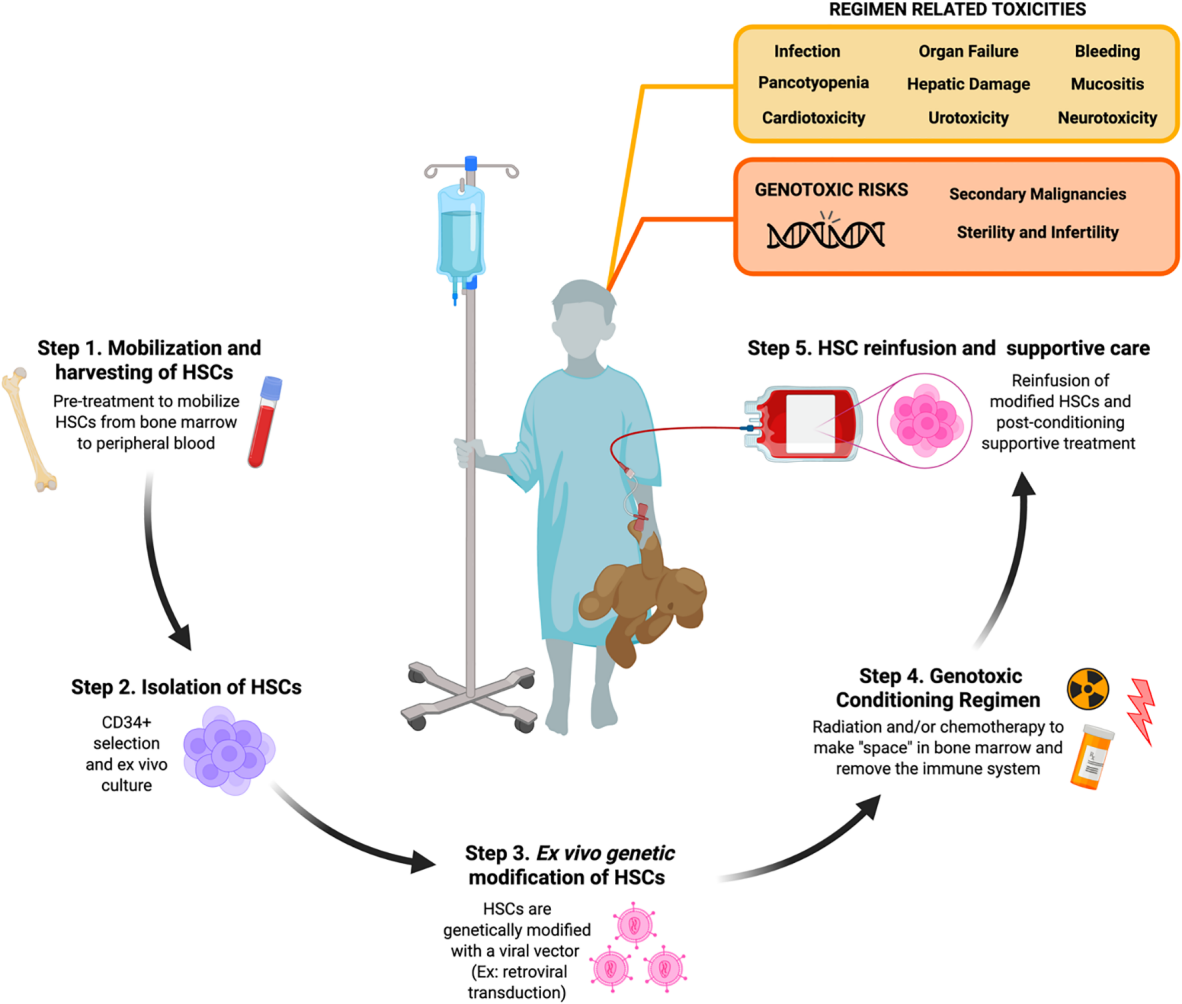
Toxicities and genotoxic risks associated with radiation and chemotherapy conditioning in patients undergoing *ex vivo* autologous hematopietic stem cell-based gene therapy. Step 1. Autologous hematopoietic stem cells (HSCs) are collected from the bone marrow (BM) niche by direct BM aspiration or peripheral blood leukapheresis of peripheral blood following mobilization pre-treatment in the patient. Step 2. Generally CD34+ cells are isolated via immunomagnetic bead section and then subject to *ex vivo* activation and culturing. Step 3. CD34+ cells are then genetically modified with a gene therapy platform like retroviral transduction. Step 4. Patients undergo conditioning with radiation and/or chemotherapy in order to clear the BM niche and suppress the patient’s immune system. Step 5. Genetically modified CD34+ cells are collected and reinfused back into the conditioned patient who is then provided post-conditioning supportive treatment until their immune system rebuilds. [Schematic created with BioRender.com].

**Figure 2:**
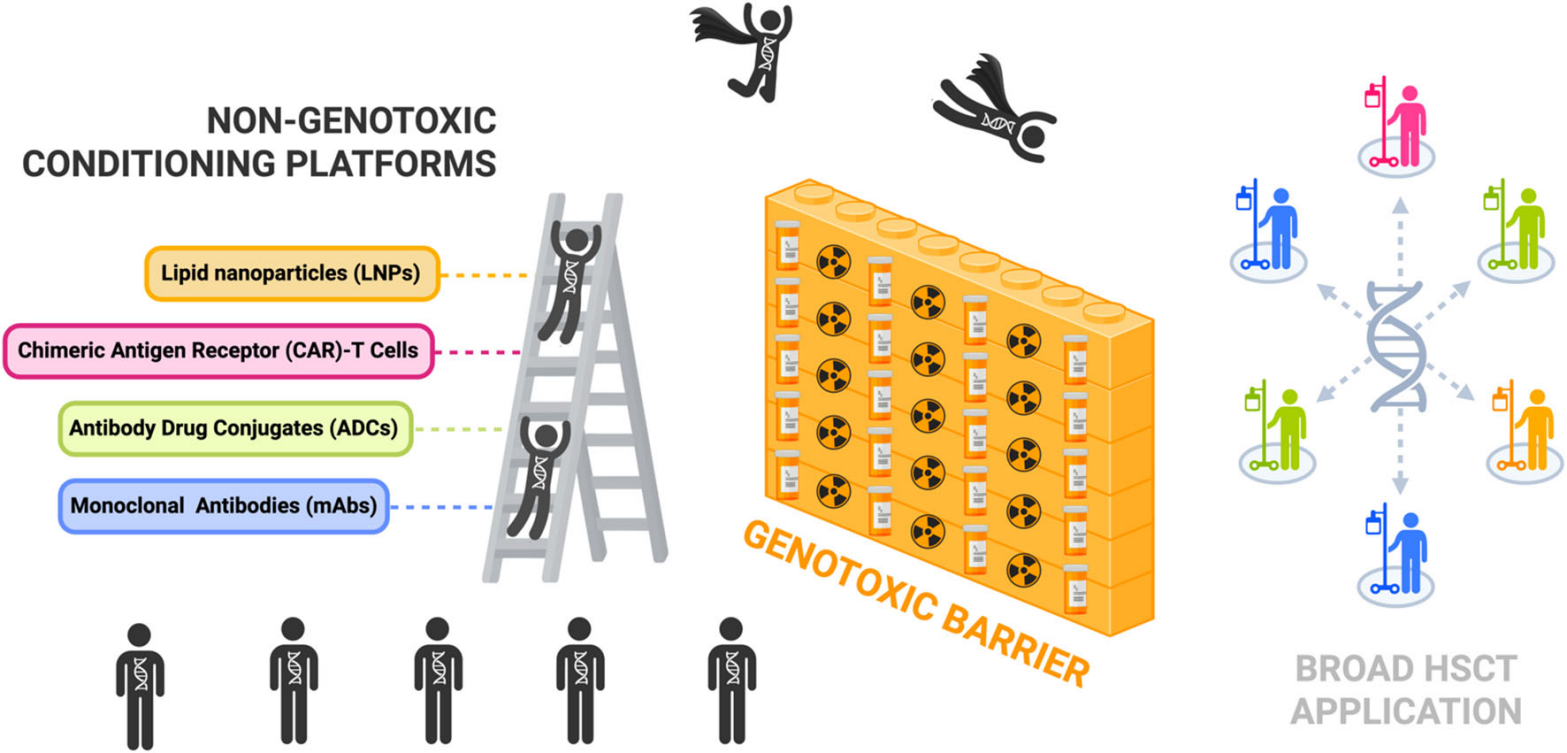
Non-genotoxic conditioning platforms can help scale the genotoxic barrier and permit broad patient access to hematopoietic stem cell transplantation for HSC-targeted gene therapies. Illustration highlights non-genotoxic conditioning platforms as an alternative to conventional conditioning with chemotherapy and/or radiation which create a genotoxic barrier limiting broader application of hematopoietic stem cell transplantation (HSCT) treatment. Key platforms currently being investigated in the field include monoclonal antibodies (mAbs), antibody drug conjugates (ADCs), chimeric antigen receptor (CAR)-T cells, and lipid nanoparticles (LNPs). [Schematic created with BioRender.com].

Unfortunately, these multi-agent MA regimens still cause pancytopenia and require long-term stem cell support after conditioning due to the profoundly cytotoxic nature of these conditioning agents. Not only do they elicit DNA damage to the BM compartment, but there is also cytotoxicity to cells and tissues not targeted in the HSC niche. MA conditioning leaves patients vulnerable to the risk of a wide range of harmful toxicities shown in **Figure 1** that include, but are not limited to, organ toxicity, infertility, severe mucositis, increased risk of infections, and secondary malignancies (12, 13). Despite the increasing use of newer RIC and NMA conditioning regimens involving these reduced doses of alkylating agents, the genotoxicity of these standard agents still acts as a barrier to limit the broader application of HSCTs for many non-malignant diseases (**Figure 2**).

**Figure 3:**
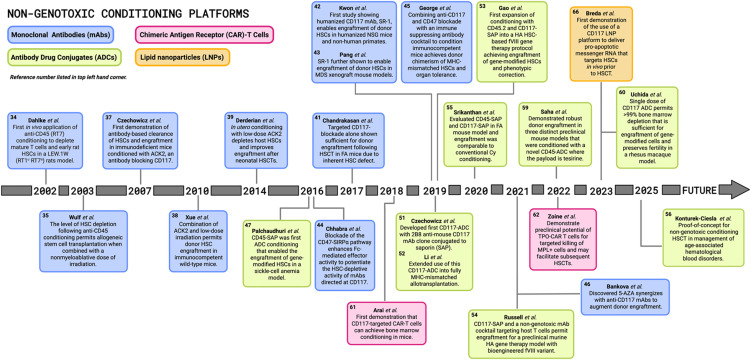
Pioneering preclinical discoveries in non-genotoxic conditioning for hematopoietic stem cell transplantations. Timeline illustrates major landmarks in the field of hematopoietic stem cell transplantation (HSCT) and HSC-directed gene therapies since the early 2000s, with various non-genotoxic conditioning platforms including monoclonal antibodies (mAbs), antibody drug conjugates (ADCs), chimeric antigen receptor (CAR)-T cells, and lipid nanoparticles (LNPs). FA, Fanconi anemia; MDS, myelodysplastic syndrome; SAP, saporin; MHC, major histocompatibility complex; HA, hemophilia A; Cy, cyclophosphamide; TPO, thrombopoietin; MPL, myeloproliferative leukemia protein; 5-AZA, 5-azacytidine. [Schematic created with BioRender.com].

There is an urgent need for the development of non-genotoxic, HSPC-targeted conditioning that can serve as an alternative solution to the genotoxic regimens currently in practice. Within the last twenty years there has been ground-breaking preclinical and clinical progress made in the HSCT field with respect to the development of non-genotoxic conditioning regimens that selectively target HSPCs and avoid serious adverse effects. Significant advances with radioimmunotherapy (RIT) compounds consisting of radionucleotides conjugated to monoclonal antibodies (mAbs) ultimately preceded the development of next generation non-genotoxic antibody- and immunotoxin-based conditioning agents (14). Recent preclinical successes shown in the timeline of **Figure 3** reveal the potential for these mAb, immunotoxin, antibody drug conjugate (ADC), chimeric antigen receptor (CAR)-T cell, and bispecific T-cell engager (BITE) approaches in the context of non-genotoxic conditioning regimens. Some of these strategies have been further optimized with antibody enhancing technologies and have advanced beyond the laboratory bench and into clinical trials where their safety and efficacy have been tested in humans. In this review we aim to consolidate these pioneering discoveries in non-genotoxic conditioning regimen development that aim to replace conventional conditioning and expand HSCT treatment for a wider range of HSC-targeted gene therapies.

## A pathway to nongenotoxic conditioning first paved by radioimmunotherapy

TBI has served as the gold-standard for conditioning regimens since the 1950s, and undoubtedly improves the success rates of transplantation by ensuring high level and long-term multilineage engraftment and reduced disease relapse. However, the extensive toxicities and severe genotoxic risks, leading to infertility and secondary malignancies, associated with TBI remain the biggest challenge limiting the transformative potential of HSCT gene therapies. Although many of these complications have been clinically well-documented for decades, ongoing research continues to further characterize the extent and mechanisms of damage done following these HSCT procedures. For example, a recent study showed allo-HSCTs leave patients susceptible to develop osteoporosis due to dysregulated mesecnchymal stem and progenitor cell (MSPC) function from elevated oxidative stress and reduced fission and mitophagy (15). Attenuating CDC42 activity *in vivo* after HSCT was able to regenerate these MSPCs to increase bone volume and trabecular bone thickness. These types of studies exploring HSCT genotoxicies are critical since they emphasize the urgent need to develop targeted conditioning platforms restricting depletion to just the HSPC niche.

**Figure 4:**
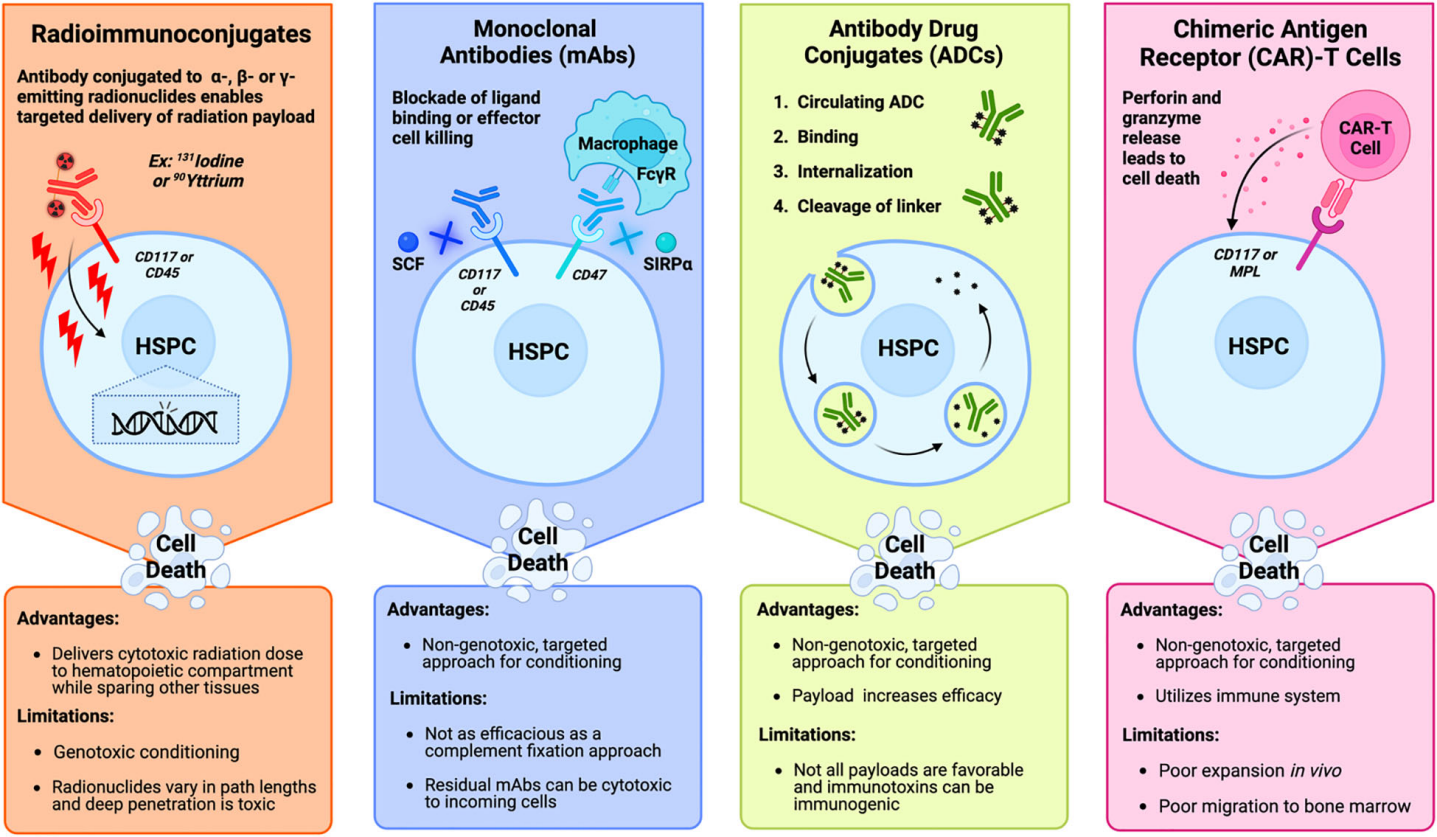
Mechanistic overview and clinical considerations of the primary approaches being studied in targeted bone marrow conditioning regimens prior to hematopoietic stem cell transplantation. Radioimmunoconjugates (far left) bind a target antigen like CD117 and CD45 on hematopoietic stem and progenitor cells (HSPCs) to deliver a cytotoxic payload of radiation. Monoclonal antibodies (middle left) deplete HSPCs by blocking ligand binding to survival receptors on HSPCs like CD117 and CD45. Blockade of CD47 prevents binding of the inhibitory molecule to increase antibody-dependent cell-mediated phagocytosis by effector cells like macrophages that recognize the Fc region of the antibody. Antibody drug conjugates (middle right) are internalized by the HSPC after binding to their target antigen. After internalization, and cleavage of the linker in the lysosome, the cytotoxic payload is released into the cytosol. Chimeric antigen receptor-T cells (far right) bind to their antigen of interest, which activates the cytotoxic effects of the T cell to lyse the HSPC through the release of perforin and granzymes. [Schematic created with BioRender.com].

The targeted transition away from TBI conditioning began in the early 1990s with the testing of radioimmunotherapy (RIT) compounds by investigators at the Fred Hutchinson Cancer Center (FHCC) as a solution to deliver cytotoxic doses of radiation to the hematopoietic compartment while simultaneously sparing or limiting toxicity to other tissues and organs. These RIT compounds, also known as radioimmunoconjugates (ROICs), consist of α -, β-, or γ- radioisotopes conjugated to either a cytolytic or neutralizing mAb (**Figure 4**). ROICs are still genotoxic, but by sparing toxicity to reproductive tissues and preserving reproductive capacity they provide a more optimal risk-benefit ratio for patients. In addition to ROIC platforms being applicable and FDA translatable, they played an instrumental role in advancing the non-genotoxic conditioning targets and strategies discussed in this review.

### CD45-directed ROICs

One of the most critical targets of choice in non-genotoxic conditioning is CD45 which is a leukocyte common antigen expressed only on the surface of nucleated hematopoietic cells. CD45 plays a vital role in their proliferation and differentiation, making it an attractive target for achieving depletion of the mature lymphoid lineage (16–18). Cytolytic radio-labelled antibodies against CD45 are among the most widely studied ROICs that have provided clinical evidence that ROIC conditioning technology can decrease disease relapse and transplant-related mortalities while still delivering targeted radiation to hematopoietic tissues.

Preclinical *in vivo* work by FHCC investigators in the early 1990s in mice and non-human primates using an anti-CD45 antibody (BC8) conjugated to the radioisotope ^31^Iodine (^131^I-BC8) was one of the earliest demonstrations of the efficacy, safety, and biodistribution of ROICs, and ultimately led to their adaptations in the clinical setting (19–22). Subsequent clinical studies revealed ^131^I-BC8 can be safely combined with fludarabine and low-dose TBI (2 Gy) in a RIC conditioning regimen prior to allo-HSCT for acute myeloid leukemia (AML) or myelodysplastic syndrome (MDS) patients over the age of 50 who would not be eligible for MA conditioning (clinicaltrials.gov identifiers: NCT02665065) (23). More recently, a phase III SIERRA trial is investigating the efficacy of Iomab-B, a next generation ^131^Iodine-anti-CD45 ROIC, when combined with fludarabine and low-dose TBI in a RIC preparative regimen prior to allogeneic HSCT in patients with active, relapsed, or refractory AML (24–26) (clinicaltrials.gov identifiers: NCT02665065).

Another notable CD45-based ROIC involves the conjugation of BC8 to the radioisotope ^90^Yttrium (^90^Y-BC8). Preclinical studies showed administration of ^90^Y-BC8 in combination with cyclophosphamide is able to replace TBI conditioning before haploidentical HSCT in a syngeneic murine leukemia model to permit long term engraftment and increase overall survival (27). A subsequent phase I clinical trial in patients with AML, MDS, or acute lymphoblastic leukemia (ALL) tested ^90^Y-BC8 in a RIC regimen with fludarabine and low dose TBI, which further showed the feasibility of using ROICs to achieve engraftment (28) (clinicaltrials.gov identifiers: NCT01300572).

One limitation of this targeted genotoxic strategy is that β emitters like ^131^Iodine and ^90^Yttrium have deep tissue penetration path lengths that could be associated with off-target toxicities (**Figure 4**). Therefore, within the RIT field, α emitters like ^213^Bismuth and ^211^Astatine, which have shorter path lengths have also been conjugated to CD45 antibodies and these particular ROICs have been extensively assessed in several *in vivo* canine models of allogeneic stem cell transplantation (29–31). Currently, phase I/II clinical trials are investigating the safety and efficacy of ^211^Astatine-CD45 mAbs in conditioning regimens prior to HSCT in both patients with nonmalignant diseases and in patients with AML, ALL, MDS or mixed-phenotype acute leukemia (clinicaltrials.gov identifiers: NCT04083183 and NCT03128034, respectively).

### FDA approved ROICs

In addition to targeting CD45, other ROICs targeting antigens such as CD20 have also been tested in clinical studies and shown promising transplant outcomes following conditioning. Out of these promising ROICS, Zevalin (^90^Yttrium ibritumomab tiuxetan) was the first CD20 targeting RIT that received FDA approval in 2002 for conditioning prior to HSCT for B-cell non-Hodgkin’s lymphoma (32). This approval was shortly followed by the FDA approval of another RIT known as Bexxar, an anti-CD20-^131^Iodine conjugate (33).

## Spectrum of preclinical non-genotoxic conditioning

### Monoclonal antibody approach

Antigen-targeted mAbs have great potential to replace non-targeted conventional chemoradiation conditioning for HSCTs due to their ability to specifically target cell types important for engraftment. Similar to how the majority of ROIC approaches focused on targeting cytolytic antigens, initial mAbs explored in the context of non-genotoxic conditioning also targeted CD45. The first *in vivo* application of an anti-CD45 (RT7^a^) mAb was performed in a LEW.1W (RT1^u^RT7^a^) rat model in the early 2000s where the effects of this anti-RT7^a^ mAb under different dosages revealed its potential to serve as an effective agent for depletion of both mature T cells and early rat HSCs (34). A study published the following year showed not only can these cytolytic CD45 mAbs achieve depletion, but this level of depletion is then able to permit allogeneic HSCT in a murine model (35). However, donor hematopoietic engraftment was only observed when the anti-CD45 mAb was combined with either a 5.5 or 8.0 Gy dose of irradiation and not when it was administered alone. The potential of CD45 mAbs was further shown in phase I/II trial testing two rat anti-CD45 mAbs (YTH24.5 and YTH 54.12) in combination with alemtuzumab, fludarabine, and low-dose cyclophosphamide in a minimal-intensity conditioning regimen prior to HSCT in patients with primary immunodeficiencies that had BM failure phenotypes (36). Engraftment in these initial CD45 mAb studies sparked a shift in the HSCT field away from the paradigm of intensive chemoradiotherapy conditioning.

Additional mAb approaches targeted CD117, also referred to as c-Kit or stem cell factor receptor (SCFR). CD117 is a dimeric transmembrane receptor tyrosine kinase that is constitutively expressed on HSPCs, but not exclusively restricted to the this particular compartment. Other CD117-dependent cell types include but are not limited to melanocytes, mast cells, germ cells, interstitial cells in the gastrointestinal tract, and certain subsets of neuronal and glial cells. Even though CD117 is expressed on other cell types it is important to note receptor expression level as well as function varies across these different tissues.

In the context of HSPCs, the interactions of CD117 with its ligand, stem cell factor (SCF also known as SCF, c-kit ligand [KL]), are essential in mediating a multitude of functions such as homing, adhesion, proliferation, maintenance, and survival of HSPCs. Taking this into consideration, initial studies tested the administration of a rat anti-mouse CD117 mAb recognizing and antagonizing CD117 as a conditioning modality for transplantation (37). The CD117 mAb led to rapid but transient depletion of >98% of endogenous HSCs in RAG2^–/–^ γc*^–/–^* immunodeficient mice. Subsequent studies showed conditioning with CD117 mAb permits stable engraftment of exogenous HSCs with donor chimerism levels up to 90% in these immunodeficient mice, but not in immunocompetent mice. However, it was later found that combining the CD117 mAb with low-dose irradiation (LD-IR) permits donor-derived HSC engraftment after congenic transplantation in immunocompetent wild-type mice (38). Furthermore, this study demonstrated the CD117 mAb and LD-IR conditioning facilitates efficient engraftment of autologous HSCs modified *ex vivo* with a lentiviral vector in X-linked chronic granulomatous disease (X-CGD) mice, demonstrating the potential for mAb conditioning in transplantations for gene therapy.

Similarly, CD117 mAb-mediated depletion of HSCs was evaluated as a fetal conditioning strategy for neonatal congenic HSCTs (39). *In utero* injections of low-dose CD117 mAb effectively eliminated host HSCs in developing mouse embryos, and HSCT on day one after birth resulted in significant levels of donor chimerism that were sustained for at least 5 months post-transplant with minimal toxicity, indicating the longevity of this conditioning regimen. Additional progress has been made in the application of non-genotoxic conditioning prior to HSC transplantation *in utero* (IUTx) as evidenced by work recently presented by the Porada group (40). A fetal sheep model tested HSC-transplanted fetuses following non-genotoxic conditioning which promoted selective depletion of recipient HSCs and successful long-term HSC engraftment. However, these specific IUTx studies did not target CD117 and instead of mAbs they employed the use of ADCs which are a non-genotoxic conditioning strategy discussed in more detail later in this review. Nonetheless, all these studies collectively highlight not only the preclinical promise of antibody-based conditioning but its potential for implementation in neonatal contexts where the expression of targets receptors outside of the hematopoietic niche could result in undesired non-HSPC cellular cytotoxicities.

Although the CD117 mAb is clearly useful in the transplant setting, it has a limited efficacy as a single agent for conditioning immunocompetent mice unless it is administered in combination with other agents. However, in a murine model of Fanconi anemia (FA) it was shown that the addition of CD117-blockade with a CD4 depleting antibody was sufficient for donor engraftment following HSCT (41). This success is due to the inherent DNA repair defects underlying the pathology of FA, which drives HSPC dysfunction and in turn progressive BM failure.

Collectively, these studies demonstrate the promise of anti-mouse CD117 mAbs for HSCTs which prompted the development of anti-human CD117 mAbs and the investigation of their applicability in more clinically relevant transplantation settings. The proof-of-concept of these anti-human CD117 mAbs as BM niche-clearing agents was demonstrated in humanized NSG mice and non-human primates using an anti-human CD117 mAb, SR-1 (42). Further studies expanded the use of SR-1 by showing it is capable of depleting MDS HSPCs and can facilitate the engraftment of normal donor human HSCs in MDS xenograft mouse models, serving as the foundation for the clinical advancement of the clinical-grade humanized SR-1, AMG191 (43), which is discussed in more detail in the clinical section of this review.

### Antibodies with enhancing technologies

CD47 is a transmembrane protein that serves as a “don’t eat me” signal via interaction with its ligand SIRPα on neutrophils and macrophages to inhibit antibody-dependent cell-mediated phagocytosis of CD47-expressing cells, such as HSPCs. Transient upregulation of CD47, a myeloid-specific immune checkpoint, is a crucial protective mechanism by which mobilized circulating HSPCs can evade macrophage destruction. The depletion of recipient HSPCs by anti-CD117 mAbs is dependent on this effector cell involvement, in addition to the blockade of SCF binding to CD117 (**Figure 4**). Therefore, the administration of CD47 antagonists or CD47 mAbs to block the CD47-SIRPα pathway will potentiate the antibody dependent cell mediated cytotoxicity potential of co-administered mAbs directed against CD117. In 2016 it was shown that preconditioning adult immunocompetent mice with the anti-CD117 antibody ACK2 in combination with an anti-CD47 antibody led to elimination of >99% host HSCs (44). Given the robust synergism between ACK2 and CD47 blockade, this approach was then combined with T cell-depleting antibodies to provide transient lymphocyte depletion during conditioning of recipients for HSC allotransplantation in a major histocompatibility complex (MHC)-mismatched model. This regimen facilitated long-term engraftment of exogenous congenic HSCs between MHC-mismatched donor/recipient pairs. The limited efficacy of naked antibodies by themselves in a non-genotoxic conditioning regimen for HSCTs can be overcome by the potential incorporation of a CD47 blockade using an anti-CD47 antibody to promote phagocytosis of target cells by immune cells. One notable example is magrolimab, also known as Hu5F9G4, which was originally explored in combination with anti-CD117 antibodies before the focus of development became centered on cancer immunotherapy. As a result magrolimab advanced to clinical testing in patients with both myeloid and solid tumors. However, some of these magrolimab clinical trials have been discontinued or placed on hold due to safety concerns observed in late-stage trials where the antibody was tested in the context of blood cancers like AML and MDS (clinicaltrials.gov identifiers: NCT04313881, NCT04778397, and NCT05079230).

In addition to blocking this dominant anti-phagocytic signal, antibody combinations that extend the use of a CD47 blockade in combination with the depletion of critical immune subsets has also been investigated. For example, one study characterized a conditioning strategy in immunocompetent mice using a six-antibody cocktail that consisted of the following mAbs: anti-CD117 to block HSC survival, anti-CD47 to promote macrophage assisted HSPC depletion, anti-CD4 and anti-CD40L to inhibit T cell mediated-rejection, anti-CD8 against cytotoxic T cells, and anti-CD122 to eliminate host natural killer cells (45). Conditioning with this six-antibody cocktail followed by transplantation enabled high donor chimerism of fully MHC-mismatched HSCs. Furthermore, fully mismatched chimeric mice were able to tolerate solid organs from the same donor following HSCTs which affirmed the mice retained their functional immunity.

CD47 blockade is not the only strategy that has been shown to display synergy with anti-CD117 mAb eradication of HSPCs. A 2021 study showed that even widely used small molecule drugs like hypomethylating agent 5-azacytidine (5-AZA), which had previously unknown effects on HSPCs, could broaden their clinical use in pre-transplantation conditioning (46). Combination of anti-CD117 and 5-AZA significantly enhanced HSPC depletion and enabled substantially higher levels of donor engraftment in immunocompetent mice.

### ADC approaches

ADCs are an attractive approach to HSCT conditioning that involve the conjugation of an antibody to either an immunotoxin or drug by a short linker molecule. One appeal of this design is it permits the delivery of cytotoxic payloads while still retaining the specificity of antibody-mediated cell targeting. Therefore, the efficacy of each ADC depends on the careful selection of the antibody, the linker, and the toxic payload. The first ADC demonstrated as a conditioning strategy for HSCTs was CD45-SAP, a CD45-targeting antibody conjugated by biotinylation to the Type I ribosome inactivating protein (RIP) saporin (SAP), that was selected from an *in vivo* HSC depletion screen, and induces cell death via apoptosis (47, 48). Unlike the prototypical ricin holotoxin that is a Type II RIP, the Type I RIPs like SAP lack lectin binding activity and in turn, a general cell entry mechanism unless conjugated to a targeted antibody (49). The study found a single dose of CD45-SAP was able to achieve 99% depletion of host HSCs and donor chimerism levels of 75-90% post transplantation in an immunocompetent mouse model of sickle cell anemia. In contrast to the irradiated controls, the administration of CD45-SAP also reduced toxicities to non-target expressing cells since ADC administration avoided neutropenia and anemia by maintaining progenitor proportions, spared the BM and thymic niches, preserved anti-fungal immunity, and enabled quicker recovery of B and T cells. Since CD45 is present on all lymphocytes the CD45-ADC did lead to profound lymphodepletion which raises the concern of opportunistic infection susceptibility. However, the translation of CD45-ADC has applications beyond allotransplantations requiring immune depletion. One notable example is in the control of autoinflammatory diseases through depletion of both the HSPCs and pathogenic immune cells. For example, Pala et al. showed CD45-ADC conditioning achieved full donor chimerism and immune reconstitution in a recombination activating gene 1 hypomorphic mouse model of combined immune deficiency with immune dysregulation (50). Collectively, all these approaches highlight the broad applications of CD45-ADCs across a range of hematological and immune-mediated disorders.

To expand the use of ADCs for HSCT conditioning where the preservation of immunity may be desired, for example in many gene therapy settings, Czechowicz et al. developed and characterized a CD117-ADC using a 2B8 anti-mouse CD117 mAb clone conjugated to SAP through biotin–streptavidin linkage (51). A single dose of the ADC led to >99% selective host HSPC depletion and enabled safe and effective HSCT of immunocompetent mice with both whole BM or purified HSCs. The downstream immunocompetent effector cells were spared due to a lack of CD117 expression, and there was a lack of neutropenia, lymphopenia, or anemia after conditioning. The ADC approach also permitted the preservation of immunity, as evidenced by the mounting of effective responses by recipients to both viral and fungal challenges. This anti-CD117-SAP conditioning approach was then tested in combination with transient immunosuppression using rapamycin and anti-CD8, anti-CD4, and anti-CD154 mAbs in order to prevent acute rejection and extend the use of the ADC into fully MHC-mismatched allotransplantation. This conditioning resulted in robust (~99%) and long-term (>1 year) hematopoietic chimerism with durable donor-specific skin allograft tolerance (52).

Based on these initial studies demonstrating the potential of ADC-mediated HSPC depletion for non-genotoxic conditioning prior to transplantation, subsequent studies aimed to expand the utility of this immunotoxin approach to the context of HSC-based gene therapies. The engraftment of gene-modified HSCs without genotoxic conditioning was first shown in HA mice using a combination of CD45.2 and CD117 ADCs conjugated to SAP in a platelet-directed HSC-based fVIII gene therapy protocol (53). Preconditioning with these agents and the supplementation of a CD8-targeting ADC was found highly effective for the engraftment of 2bF8 lentivirus (LV)-transduced HSCs, resulting in sustained therapeutic platelet fVIII expression and phenotypic correction as determined by a needle induced knee joint injury and a tail-bleeding assay. Conditioning with CD117-SAP coupled with the administration of a non-genotoxic mAb cocktail targeting host T cells was investigated in a different preclinical murine HA gene therapy model to demonstrate successful endogenous HSPC depletion and transient immunosuppression, respectively (54). This strategy provided high-level and long-term engraftment of HSCs genetically modified *ex vivo* using a recombinant LV encoding a bioengineered fVIII variant, termed ET3. No immunological rejection was observed, and phenotypic correction was achieved following transplantation of these ET3-modified donor HSCs.

In addition to gene therapy for hemophilia, these ADCs have also been studied in non-genotoxic conditioning for other hematologic diseases. One group evaluated the conditioning ability of CD45-SAP and CD117-SAP in a well-established mouse FA model (55). These ADCs facilitated effective multi-lineage engraftment of FA-heterozygous cells that was comparable to conventional cyclophosphamide conditioning. Furthermore, Konturek-Ciesla et al. have recently shown application of non-genotoxic conditioning with CD45-SAP to introduce young HSCs into aged hosts as a prophylactic tool to prevent onset of age-associated hematological disorders. Aged BM microenvironments can interfere with HSC engraftment, and this study along with another by the Weissman group showing myeloid-biased HSCs drive the aged phenotype, both highlight the importance of assessing ADC efficacy in the context of different aged recipients where the homing efficiency, immunity, and inflammation may vary (56, 57). Collectively, these different preclinical studies establish strong proof-of-concept towards the translation of these non-genotoxic conditioning platforms for allogeneic transplantations as well as gene therapy strategies.

### Immunotoxin payload considerations for ADCs

There are several considerations that must be noted for the selection of immunotoxins such as SAP for the payload. The clinical translation of many of these ADCs that feature RIPs is limited by the fact that many of these immunotoxins such as SAP are well known to induce immune responses with neutralizing anti-toxin antibodies (49). Therefore, studies are investigating the efficacy of additional ADC designs with various payloads. For example, Pearse et al. conjugated an anti-CD117 antibody to amanitin that is derived from the *Amanita phalloides* species of mushroom. Interestingly, this is also a Type I RIP and was the only toxin from their screen able to achieve >90% depletion of human HSPCs in humanized NSG mice (58). They followed these encouraging results in a rhesus macaque model and showed >99% depletion of HSPCs while preserving BM lymphocytes.

More recently, Saha et al. reported robust donor engraftment in three distinct preclinical mouse models that were conditioned with a novel CD45-ADC conjugated to a tesirine payload instead of a RIP (59). Tesirine is an alkylating pyrrolobenzodiazepine dimer with antimitotic and cytotoxic activity that achieves targeted HSC depletion by interfering with DNA interstrand crosslinking which leads to cell cycle arrest followed by death. Uchida et al. also utilize a tesirine payload in their CD117 ADC which permitted engraftment of gene-modified cells and preserved fertility in a rhesus macaque lentiviral gene therapy model for hemoglobinopathies (60). Even though these results suggest certain toxins and their mechanisms are more favorable for targeted HSC killing when compared to other drug conjugates, more studies are needed to further elucidate the potency of these immunotoxins on the stem cell compartment.

### CAR-T cell, BiTE, and LNP approaches

Chimeric antigen receptor T (CAR-T) cells are T cells genetically modified to express a recombinant receptor targeting the engineered T cell to a specific antigen. Typically, CAR antigen specificity is mediated through a single chain variable fragment (scFv) that consists of a variable heavy and light chain of a monoclonal antibody fragment connected by a peptide linker. However, natural receptor- or ligand-based CAR designs are also being explored. Binding of the CAR to the antigen of interest subsequently activates the cytotoxic activity of the T cell through the CAR cytosolic CD3ζ domain, bypassing engagement of the major histocompatibility complex. As shown in **Figure 4**, the release of perforin and granzymes from the activated CAR-T will then induce specific and rapid target cell lysis. Because the genetically engineered CAR-T cells are only activated upon external binding to the target antigen, it is an appealing strategy to apply in the context of HSPC conditioning.

The first demonstration that CAR-T cells can be used for BM conditioning was reported in 2018 by assessing HSPC depletion with anti-CD117 directed CAR-T cells and subsequent engraftment in immunocompetent mice (61). The study first showed mouse CD117 CAR-T cells can effectively bind and kill CD117+ cells *in vitro*. Subsequent studies *in vivo* revealed treating mice with a low-dose of cyclophosphamide in combination with the CD117 CAR-T cells permits donor chimerism of around 20-40%. Interestingly, they also found mouse CD117 CAR-T cells required genetic engineering to overepress the chemokine receptor 4 (CXCR4) and achieve migration of the anti-CD117-CAR-T cells to the BM. A limitation of this study is that cyclophosphamide is genotoxic; this approach will require more optimization to become nongenotoxic. Nonetheless, this important finding highlights the general principle that co-expression of trafficking receptors can enhance the targeting of CAR-T cells to desired anatomic locations to augment the effectiveness of targeted cell killing.

Although targeting HSCs using scFV-based CAR-T cells shows promise, novel strategies for applying CAR-T cell therapy to non-genotoxic conditioning for HSCTs were developed. For example, it was recently shown that anti-HSPC directed CAR-T cells could be generated using a ligand binding domain targeting the antigen thrombopoietin (TPO) (62). These TPO-CAR-T cells engage the myeloproliferative leukemia protein (MPL) receptor that possesses an integral role in survival signaling, quiescence, and DNA repair of both normal HSPCs and megakaryocytic AML cells. TPO is an ideal target for HSPC-directed conditioning regimens due to its minimal expression within the non-hematopoietic compartment. Zoine et al. demonstrated in AML xenograft models that TPO-CAR-T cells are cytotoxic against the MPL+ fraction of leukemia cells in the BM compartment. Subsequent studies are needed to evaluate the preclinical potential of scFV based CAR-T cells like these in the context of HSCTs.

Similar to CAR-T cells, there has been a recent rise in the development and investigation of bispecific T cell engagers (BiTEs). BiTEs are a class of artificial bispecific mAbs that permit simultaneous targeting of two different antigens, i.e. a tumor antigen and a T cell antigen such as CD3. This design allows BiTEs to redirect T cells toward tumor cells, and as such they are predominantly being developed as anti-cancer therapeutics. However, recently a CD34-CD3 BiTE was shown to achieve T-cell-mediated depletion of CD34+ HSCs and CD34+ blasts from AML patients (63). Subsequent application in humanized AML xenograft models confirmed the *in vivo* efficacy of the CD34-specific BiTE. Another BiTE that could be repurposed in the context of a conditioning regimen would be a CD117-specifc BiTE that has been shown to induce selective T cell-mediated depletion of CD117-expressing healthy HSPCs and residual AML or MDS cells (64). Lastly, it was shown that a BiTE targeting Fms-like tyrosine kinase 3 (FLT3), which has restricted expression to HSCs, facilitates *in vivo* elimination of both normal HSPCs and AML cells in a humanized mouse model (65). All these preliminary studies highlight the promise for the application of CD34, CD117, or FLT3-specific BiTEs in the context of HSCT conditioning.

However, CAR-T cells and BiTEs are not the only nonconventional modalities being investigated for their potential in non-genotoxic conditioning. A study by Breda et al. utilized lipid nanoparticles (LNPs) functionally coupled to CD117 antibodies to provide transient delivery of messenger RNA (mRNA) to HSPCs (66). Not only did this CD117/LNP-platform permit *in vivo* HSC engineering through effective delivery of prokaryotic site-specific Cre recombinase, but it was also able to deliver pro-apoptotic p53 up-regulated modulator of apoptosis (PUMA) mRNA to deplete mouse HSPCs in the BM niche. Although the levels of donor cell engraftment following a whole BM transplant in CD117/LNP-PUMA conditioned recipient mice were low, this conditioning shows the promise for the use of LNPs as a platform for delivering mRNA to either deplete the HSPC compartment in preparation for HSCT with *ex vivo* genetically engineered HSPCs or to directly *in vivo* engineer HSPCs residing in the BM niche.

All non-genotoxic conditioning platforms have their own set of limitations though and LNP delivery systems are no exception. Among the several drawbacks of LNPs is their low drug load efficiency and the considerable gap in knowledge regarding their long-term immunogenicity (67). Using LNPs to target niche populations like HSPCs also creates complications due to the need for stem cell harvest, culture, or mobilization depending on the context of either *in vivo* or *ex vivo* mRNA delivery. Nonetheless, these barriers should not be deterrents for the exploration of LNPs in conditioning and gene therapy contexts. It is encouraging to see that groups like Shi et al. are already optimizing these platforms to allow for higher delivery to HSPCs (68).

### Mobilization approaches

Another approach that merits discussion is the addition of mobilizing agents into these conditioning regimens to enable long-term engraftment and multilineage differentiation. Mobilization agents like plerixafor (PX) are frequently used clinically to mobilize HSPCs out of the BM niche as a source of cells prior to HSCT, but there is now growing preclinical evidence demonstrating their application in genotoxicity-free conditioning strategies. For example, Omer-Javed et al. show *ex vivo* cultured HSPCs have rescued CXCR4 expression and a competitive migration advantage over PX mobilized HSPCs for engraftment in a mouse model of hyper IgM syndrome (69). More recently Ojeda-Perez et al. combined anti-CD117 and anti-CD47 antibodies with PX to achieve effective HSPC depletion and multilineage engraftment in wildtype and RAG2^–/–^ mice, but also increase survival in PKD mice (70). Although both of these studies offer extensive and promising preclinical evaluation, the use of PX as a conditioning therapy remains inconclusive in the limited clinical literature that is available (71–73). Even though this approach is still in early stages of development, it is an innovative alternative that warrants continued investigation.

## Targeted conditioning in clinical care

### Antibody-based strategies in the clinic

The preclinical studies discussed in the former section were instrumental in helping advance some of these novel mAb, immunotoxin, and CAR-T cell conditioning approaches into clinical testing. Out of the mAbs the humanized antibody JSP191 targeting CD117, also known as briquilimab and formerly called AMG191, has advanced the furthest in Jasper-sponsored phase I/II clinical trials. For example, it has already been evaluated in more than 120 healthy volunteers and patients with SCID or AML/MDS (clinicaltrials.gov identifiers: NCT02963064 and NCT04429191). Although there are limited updates regarding the status of these trials, the preliminary reports of the SCID study do show initial clinical proof-of-concept benefits of targeted single-agent JSP191 conditioning which enables donor HSC engraftment and immune reconstitution (74, 75). In addition to these studies testing JSP191 as a single agent, an additional FA clinical trial is investigating JSP191 in combination with anti-thymocyte globulin, cyclophosphamide, fludarabine, and rituximab (clinicaltrials.gov identifiers: NCT04784052). As we discussed in the introduction, depending on the clinical setting there can be many benefits to the use of a multi-agent conditioning regimen. Therefore, it is promising to see the testing of the inclusion of non-genotoxic conditioning platforms like mAbs alongside these established agents where they have the potential to augment HSCT outcome.

ADC administration strategies have also progressed to clinical trials in recent years. For example, Magenta Therapeutics opened phase I/II clinical trials in late 2021 to evaluate the safety, efficacy, and pharmacokinetics/pharmacodynamics of targeted conditioning using an anti-CD117-amanitin ADC named MGTA-117 ADC (clinicaltrials.gov identifier: NCT05223699). MGTA-117 revealed in preclinical studies that it can achieve effective depletion of CD117+ HSCs and leukemic blasts (76). However, the clinical studies were conducted in patients with relapsed/refractory AML and MDS which is notably a different transplant setting than the original indication of the ADC. In early 2023, the phase I/II dose escalation trials were halted due to pulmonary toxicity. This discontinuation is not indicative of a failure of the antibody though since it was only tested in the context of relapsed AML/MDS. Therefore, its safety and efficacy in the transplant setting for which it was designed remains unknown.

### Technologies to overcome persistence of circulating antibodies

It should be noted MGTA-117 was engineered to lack fragment crystallizable receptor (FcRn) binding activity, and in turn had a half-life that was approximately half of a non-modified antibody (76). This strategy is used in an attempt to overcome one of the prime limitations of mAb and ADC approaches for HSCT conditioning, which is that any residual mAbs or ADCs in circulation at the time of transplant may interfere with the engraftment of donor HSCs (**Figure 4**). Other potential strategies to achieve rapid ADC clearance and limit cytotoxicity to incoming donor cells that are being tested preclinically include the use of enzymatic cleavage of IgG antibodies or the direct targeting of FcRn. For example, regarding enzymatic digestion of IgG, IdeS is a proteinase derived from *Streptococcus pyogenes* that is a cysteine endopeptidase with a high degree of substrate specificity for IgG (77). Regarding the targeting of FcRn, this strategy prevents IgG antibody recirculation and decreases the systemic half-life. Both strategies decrease the circulating plasma half-life of antibodies, and thereby decreases the effects of the antibody on transplanted cells, which can potentially improve BM engraftment following mAb or ADC conditioning.

## Conclusions

Recent clinical successes have revealed the power and potential of autologous HSC-directed gene therapy as a curative treatment modality for a variety of malignant and nonmalignant hematopoietic diseases. Even though these results are encouraging, the conventional use of DNA-damaging, genotoxic conditioning agents prior to transplant continues to limit broad clinical impact of HSCT gene therapy. Conventional conditioning with irradiation and alkylating chemotherapeutics is non-targeted and creates an increased risk of sterility, infection, and the development of secondary malignancies. There is no denying that there has been important progress in terms of conditioning patients with intermediate doses of standard radiation and chemotherapy agents or even by using RIT compounds as an alternative to complete myeloablation. However, the long-term pathophysiological implications of any cells that survive exposure to genotoxic agents during these RIC regimens remains unknown and its risk-benefit ratio still poses a challenge for the applicability of HSC-targeted gene therapies for the treatment of younger patients.

One alternative to avoid conventional conditioning is to simply use direct *in vivo* gene therapy as opposed to *ex vivo* gene therapy, and there is a recent trend in the field where this strategy is already being tested in the clinic (78). Companies are also adopting this transition, but even though this trend is promising, *ex vivo* gene therapy stands at the forefront and will likely remain relevant for many contexts where *in vivo* gene therapy will simply not be applicable. For example, lentiviral gene therapy for hemophilia A has proven to be successful in clinical trials but this is a disease context in which *in vivo* gene therapy contexts would likely induce inhibitors (79). Therefore, the development of non-genotoxic conditioning regimens that selectively target HSPCs continues to be a high-priority translational objective that will remove the acute and long-term toxicities associated with conventional conditioning.

Within this review we have synthesized the current preclinical and clinical advancements supporting the use of non-genotoxic strategies in conditioning regimens prior to HSCT. These studies serve as proof-of-concept that antigen-targeted mAbs, ADCs, and immunotoxins can be used as efficacious BM niche-clearing agents that deplete donor cells while preserving BM architecture and permitting engraftment of gene-modified HSCs. Promising results have even ushered in a new era investigating the use of T cell redirection strategies with CARs and BiTEs within the context of BM depletion. However, as the field of non-genotoxic conditioning continues to advance, there are a variety of challenges that are likely to manifest as made evident during Magenta Therapeutic’s clinical trials for MGTA-117. Currently HSPC targets for non-genotoxic conditioning like CD117 are based on the steady state expression of receptors, but an important area of research should be assessing how the expression of these targets and the stability of selected immunotoxins like amanitin is altered within disease-specific contexts. Another inherent challenge for non-genotoxic conditioning is the persistence of circulating antibodies that have the potential to interfere with incoming gene-modified HSCs at the time of transplant. Within the field, Fc engineering has already been successfully used as a method to decrease antibody half-life, and it is exciting to witness the further investigation of other alternatives including, but not limited to, the use of FcRn inhibitors and antibody cleavage strategies. Although conventional conditioning with chemoradiation remains the gold standard of our current generation for autologous HSCT, based on the pioneering discoveries in this review it is evident that targeted non-genotoxic conditioning will ultimately expand the utility of this potentially curative gene therapy platform to a wider range of patients in the next generation.
